# Type 1 Diabetes Patients With Different Residual Beta-Cell Function but Similar Age, HBA1c, and Cardiorespiratory Fitness Have Differing Exercise-Induced Angiogenic Cell Mobilisation

**DOI:** 10.3389/fendo.2022.797438

**Published:** 2022-02-11

**Authors:** Guy S. Taylor, Andy Shaw, Jadine H. Scragg, Kieran Smith, Matthew D. Campbell, Timothy J. McDonald, James A. Shaw, Mark D. Ross, Daniel J. West

**Affiliations:** ^1^ Population Health Sciences Institute, Newcastle University, Newcastle upon Tyne, United Kingdom; ^2^ Nuffield Department of Primary Care Health Sciences, University of Oxford, Oxford, United Kingdom; ^3^ Faculty of Health Sciences and Wellbeing, University of Sunderland, Sunderland, United Kingdom; ^4^ Leeds Institute of Cardiovascular and Metabolic Medicine, University of Leeds, Leeds, United Kingdom; ^5^ National Institute for Health Research Exeter Clinical Research Facility, University of Exeter Medical School, Exeter, United Kingdom; ^6^ Academic Department of Blood Sciences, Royal Devon and Exeter NHS Foundation Trust, Exeter, United Kingdom; ^7^ Translational and Clinical Research Institute, Newcastle University, Newcastle upon Tyne, United Kingdom; ^8^ Newcastle Centre for Diabetes Care, Newcastle upon Tyne Hospitals NHS Foundation Trust, Newcastle upon Tyne, United Kingdom; ^9^ School of Applied Sciences, Edinburgh Napier University, Edinburgh, United Kingdom

**Keywords:** residual beta-cell function, haematopoietic progenitor cells, endothelial progenitor cells, exercise, exercise-induced mobilisation

## Abstract

**Background:**

Many individuals with type 1 diabetes retain residual beta-cell function. Sustained endogenous insulin and C-peptide secretion is associated with reduced diabetes related complications, but underlying mechanisms remain unclear. Lower circulating numbers of endothelial and hematopoietic progenitor cells (EPCs and HPCs), and the inability to increase the count of these cells in response to exercise, are also associated with increased diabetes complications and cardiovascular disease. It is unknown whether residual beta-cell function influences HPCs and EPCs. Thus, this study examined the influence of residual beta-cell function in type 1 diabetes upon exercise-induced changes in haematopoietic (HPCs) and endothelial progenitor cells (EPCs).

**Methods:**

Participants with undetectable stimulated C-peptide (n=11; Cpep_und_), 10 high C-peptide (Cpep_high_; >200 pmol/L), and 11 non-diabetes controls took part in this observational exercise study, completing 45 minutes of intensive walking at 60% $\dot{V}O_{2peak}$ . Clinically significant HPCs (CD34^+^) and EPCs (CD34^+^VEGFR2^+^) phenotypes for predicting future adverse cardiovascular outcomes, and subsequent cell surface expression of chemokine receptor 4 (CXCR4) and 7 (CXCR7), were enumerated at rest and immediately post-exercise by flow cytometry.

**Results:**

Exercise increased HPCs and EPCs phenotypes similarly in the Cpep_high_ and control groups (+34-121% across phenotypes, p<0.04); but Cpep_und_ group did not significantly increase from rest, even after controlling for diabetes duration. Strikingly, the post-exercise Cpep_und_ counts were still lower than Cpep_high_ at rest.

**Conclusions:**

Residual beta-cell function is associated with an intact exercise-induced HPCs and EPCs mobilisation. As key characteristics (age, fitness, HbA1c) were similar between groups, the mechanisms underpinning the absent mobilisation within those with negative C-peptide, and the vascular implications, require further investigation.

## 1 Introduction

Residual beta-cell function can persist in individuals with established type 1 diabetes, with between 35% to 80% of participants estimated to have detectable function at >5 years post diagnosis (1, 2). Sustained endogenous insulin and C-peptide secretion appears protective against complications in type 1 diabetes (3–7), however the underlying mechanisms are unclear. It is likely that the improved glycaemic control that is associated with residual beta-cell function (5, 7–10) results in reduced vascular damage, with hyperglycaemia acknowledged as the principal cause of microvascular complications (11). However, significant reductions in HbA1c and time spent in hyperglycaemia only occur at high beta-cell function (C-peptide ~200 pmol/L) (4, 9, 12), while even very low levels of beta-cell function may offer protection against diabetes-related complications (C-peptide >10 pmol/L) (5, 7, 13). It is unclear how beta-cell function may protect against complications independently of reducing hyperglycaemic induced damage.

There is a plethora of evidence that haematopoietic stem/progenitor cells (HPC), and endothelial progenitor cells (EPCs) have the potential to stimulate vascular repair (14–16). Identifiable by flow cytometry, HPCs are mononuclear cells in the peripheral blood expressing markers of haematopoiesis, such as CD34 (17), while EPCs have additional endothelial markers such as VEGFR2 (18). Compared to matched healthy controls, individuals with type 1 diabetes have reduced circulating counts of both HPCs and EPCs (19–24), with lower resting counts associated with increased occurrence of diabetes complications and cardiovascular disease (CVD) (19, 20, 25, 26). Recently, it has been suggested that exercise-induced mobilisation of angiogenic cells may be a predictor of future complications. Having no exercise-induced mobilisation was a stronger risk factor future CVD occurrence in patients with coronary artery disease than resting counts (27), while pre-operative exercise-induced mobilization is correlated with post-operative complications after major thoracic surgery (28). Research into exercise-induced mobilisation within type 1 diabetes has found mixed results, with either a complete lack of exercise-induced increase (29, 30), or an attenuated increase compared to matched controls (31).

No previous research has directly explored whether residual beta-cell function influences HPC and EPC counts. However, studies into individuals with extreme duration type 1 diabetes (>50 years), who appear to have endogenous factors that can reduce and prevent diabetes complications, may offer clues (32). Compared to the general type 1 diabetes population, this “Medalist group” are more likely to retain a higher beta-cell function (33). Additionally, they have higher circulating counts of angiogenic cells, comparable to the numbers found in matched non-diabetes controls (26). Therefore, the aim of this study was to explore whether residual beta-cell function in type 1 diabetes influences HPC and EPC mobilisation in response to exercise, and to compare against non-diabetes controls.

## 2 Materials and Methods

### 2.1 Study Design

This analysis in individuals with type 1 diabetes ≥3 years’ duration, was a pre-specified secondary outcome of an observational study comparing exercise-induced changes with non-diabetes controls (31) (registry:ISRCTN63739203). Recruitment, consent, research design; including blood glucose management, and ethical approval has been described in detail elsewhere (31). Briefly, participants with type 1 diabetes had a confirmed clinical diagnosis; a diabetes duration ≥ 3 years; age 18–65 years; HbA1c < 86 mmol/mol (10.0%); and absence of diabetes-related complications, including any history of neuropathy or kidney issues, apart from non-proliferating retinopathy. For the non-diabetes participants, eligibility criteria comprised being aged between 18 and 65 years, non-smoker, and free from any history of chronic diseases. Participants provided written informed consent and the study was approved by the NHS HRA North East Tyne & Wear South Research Ethics and Newcastle University Ethics Committees (code:16/NE/0192).

All participants attended the Newcastle NIHR Clinical Research Facility (CRF) to determine peak oxygen uptake ( $\dot{V}O_{2peak}$ ) and to complete the main trial. Participants with type 1 diabetes also completed a mixed-meal tolerance test visit. Recruitment and data collection ran from October 2016 to September 2019.

### 2.2 Mixed-Meal Tolerance Test

Participants attended the CRF after an overnight fast, as previously described (10). Briefly, participants were given 240 mL Fortisip (Nutricia, Trowbridge, U.K.) (360 kcal, 14.4 g protein, 13.92 g fat, and 44.16 g carbohydrate) to drink within 2 min (34). Blood samples were drawn at baseline and every 30 min after consumption, up to 180 minute. Peak serum C-peptide was used to stratify participants. Eleven with undetectable C-peptide (Cpep_und_ <3 pmol/L) and 10 with high residual C-peptide (Cpep_high_ >200 pmol/L) were included in the current analysis.

### 2.3 $\dot{V}O_{2peak}$ Trial

Participants attended the CRF exercise lab to determine $\dot{V}O_{2peak}$ and screen for any cardiac abnormalities. Participants underwent a modified 12-lead resting and exercising electrocardiogram as well as completing a maximal graded walking treadmill (Valiant 2 CPET; Lode, Groningen, the Netherlands) Bruce protocol test (35) to determine $\dot{V}O_{2peak}$ .

### 2.4 Main Trial

Participants attended the CRF exercise lab after an overnight fast. Participants with type 1 diabetes maintained their normal basal insulin regimen. If they experienced a hypoglycemic event overnight prior to the study visit, the visit was cancelled and reorganised. If upon waking blood glucose was > 10 mmol/L, they were instructed to have a small corrective bolus of rapid-acting insulin (≤ 2 units).

Cannulation was performed into an antecubital vein, and after discarding the initial 4mL drawn to avoid contamination of mature endothelial cells disturbed from the punctured vein, resting blood samples were drawn. A 10 mL EDTA vacutainer (Becton, Dickinson and Company, New Jersey, USA) was collected at rest and, immediately post-exercise for flow cytometry enumeration. An additional 4 mL EDTA Vacutainer was for analysis of HbA1c at the Newcastle Clinical Laboratory. Capillary blood was collected at all-time points and analysed by a HemoControl analyser (EKF, Cardiff, UK) to determine haematocrit and haemoglobin concentration.

Participants consumed a 30 g carbohydrate snack (Belvita, Mondelēz International, USA) immediately after baseline blood draws and remained rested for 20 min. The exercise bout comprised of incline walking (9.0±4.6% at 4.9±1.0kph) for 45 minutes at 60% $\dot{V}O_{2peak}$ with respiratory parameters (Metalyzer® 3B-R3 CPET, Cortex, Leipzig, Germany) recorded throughout. If $\dot{V}O2$ was >10% different than target $\dot{V}O2$ at 10 and 30 min into the exercise, the gradient was adjusted. Participants with type 1 diabetes had a target capillary blood glucose >7 mmol/L for the duration of the exercise. Six individuals given 10 g of additional carbohydrates, administered *via* a glucose drink. At exercise completion, blood samples were immediately drawn from the cannula.

#### 2.4.1 Flow Cytometry Enumeration of Hematopoietic and Endothelial Progenitor Cells

Angiogenic cells were enumerated by flow cytometer (LSRFortessa™ X20; BD Biosciences, USA) within 4 hours of blood draw. Whole blood collected in EDTA vacutainer (Becton, Dickinson and Company, UK) was incubated with anti-CD34 FITC, anti-VEGFR2 APC, anti-CD45 BV421, anti-CXCR4 APC-Cy7, and anti-CXCR7 PE (BioLegend, USA) in a Trucount™ tube (BD Biosciences, USA) with red blood cell lysis buffer (BD PharmLyse^TM^, BD Biosciences, USA). Samples were analysed for 45 minutes or until 500,000 CD45^+^ events had been enumerated.

Flow cytometry files were analysed using FCS Express 7 (*De Novo*, California, USA). Counts of HPCs (CD34^+^) and EPCs (CD34^+^VEGFR2^+^) and subsequent cell surface expression of chemokine receptor 4 (CXCR4) and 7 (CXCR7) were converted to cells/mL using BD Trucount™, and adjusted for changes in blood volume [further methodology details available elsewhere (31)]. Briefly, these phenotypes were chosen as low circulating CD34^+^ HPCs and CD34^+^VEGFR2^+^ counts are proven as risk factors to future adverse cardiovascular outcomes and death (25, 27). Surface expression of CXCR4 and CXCR7 gives insight into how these cells respond to a stimuli such as exercise (31), characterising their ability to home to hypoxic environments, and may be a better predictor of mortality than HPCs and EPCs alone (27).

### 2.5 Statistical Analysis

Analysis was performed using SPSS-24.0 (IBM CORP, Armonk, USA) and GraphPad Prism 8.0.1 (San Diego, USA). Normality and outliers were assessed, with skewed data transformed. A Two-Way Mixed Model ANOVA was used to examine the interaction on cell numbers over time (resting and post exercise) and between groups (Cpep_und_, Cpep_high_ and non-diabetes controls), with statically significant effects examined by Bonferroni *post-hoc* tests. An ANCOVA, adjusting for HbA1c, age or $\dot{V}O_{2peak}$ was used to compare all groups across time. An ANCOVA, adjusting for diabetes duration, was used to compare the diabetes groups across time. Data are presented as means ± standard deviation. Statistical significance was set at p≤0.05.

## 3 Results

Age, BMI and $\dot{V}O_{2peak}$ were comparable between all groups (**Table 1**). The Cpep_high_ group had significantly shorter duration diabetes (11.4±6.5 years) than Cpep_und_ group (26.2±13.9 years, p=0.006), but similar HbA1c (56±10 vs 61±10 mmol/mol, p=0.486)

**Table 1:** Participant demographic data.

Grouping	Cpep_und_	Cpep_high_	Non-diabetes Control
***N***	11	10	11
***Male/Female***	5/6	5/5	5/6
***Age (Years)***	39.64 ± 11.24	36.30 ± 11.11	38.00 ± 12.06
***HbA1c (mmol/mol)***	60.64 ± 9.96	56.50 ± 10.00	33.18 ± 1.99 *†
***(%)***	7.7 ± 0.9	7.3 ± 0.9	5.2 ± 0.2 *†
***BMI (kg/m^2^)***	25.66 ± 3.26	25.66 ± 4.05	25.55 ± 4.38
$\dot{V}O_{2peak}$ ***(ml/kg/min)***	35.23 ± 7.90	38.06 ± 10.22	37.26 ± 9.64
***Cholesterol (mmol/L)***	4.5 ± 1.1	4.0 ± 0.7	4.8 ± 0.8
***HDL Cholesterol (mmol/L)***	1.7 ± 0.4	1.5 ± 0.3	1.5 ± 0.4
***Triglycerides(mmol/L)***	0.8 ± 0.2	0.7 ± 0.3	1.1 ± 0.5
***Age At Diagnosis***	13.27 ± 4.50	25.10 ± 8.20 *	-
***Range (Years)***	8 to 24	13 to 35	
***Duration Of Diabetes***	26.18 ± 13.91	11.40 ± 6.45 *	-
***Range (Years)***	10 to 47	3 to 20	
***Method Of Control (MDI/CSII)***	5/6	6/4	-

Data presented as mean ± SD. * represents a significant differences compared to Cpep_und_, ^†^represents a significant differences compared to Cpep_high_. P value from one-way ANOVA and independent samples t-test.

Participants exercised at 58% of $\dot{V}O_{2peak}$ , with no differences between groups (p=0.405). There were no episodes of hypoglycemia (<3.9 mmol/L). Exercise was a significant stressor, inducing a mean energy expenditure of 1558±511 kJ (373±122 kcal), with no differences between groups (p=0.809). There was no missing data.

### 3.1 Exercise-Induced Change in HPCs and EPCs

**Figure 1:**
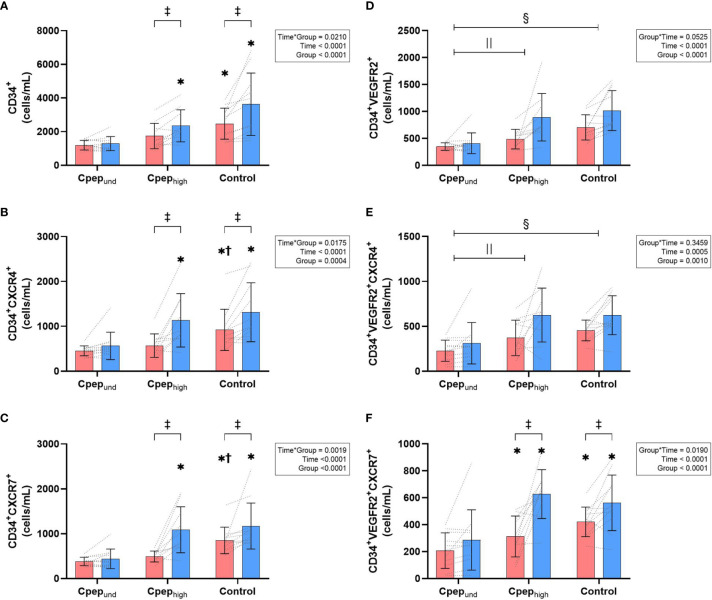
Mean (± SD) group and individual time course changes from rest (red bars) to immediately post-exercise (blue bar) for absolute count (cells/mL) of CD34^+^ HPCs **(A)**, CD34^+^ HPCs expressing CXCR4^+^ **(B)**, and CD34^+^ HPCs expressing CXCR7^+^ **(C)**, as well as CD34^+^VEGFR2^+^ EPCs **(D)**, CD34^+^VEGFR2^+^ EPCs expressing CXCR4^+^ **(E)**, and CD34^+^VEGFR2^+^ EPCs HPCs expressing CXCR7^+^ **(F)**. Data were analysed by a two-way mixed-model ANOVA. Where a statistically significant interaction effect exists - * represents a significant differences compared to Cpep_und_ at that timepoint, ^†^ represents a significant differences compared to Cpep_high_ at that timepoint, ^‡^ represents a significant differences from rest to immediately post-exercise. Where a statistically significant interaction effect did not exists, 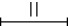 represents a significant group differences between Cpep_und_ and Cpep_high_, 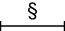 represents a significant group differences between Cpep_und_ and non-diabetes controls.

There was a significant group*time interaction for HPCs, CXCR4^+^ HPCs and CXCR7^+^ HPCs phenotypes, as well as the CXCR7^+^ EPCs (**Figures 1A–C, F**). Resting counts were highest for participants without diabetes, lower for Cpep_high_ group (CXCR4^+^ and CXCR7^+^ HPCs significantly so) and lowest in those with undetectable C-Peptide (all phenotypes significantly lower than controls, CXCR7^+^ EPCs significantly lower than Cpep_high_). HPCs phenotypes and CXCR7+ EPCs increased significantly from rest to post-exercise in the Cpep_high_ and non-diabetes control groups (34-121% increase), with no significant changes in the Cpep_und_ group (8-38% increase). For EPCs and CXCR4^+^ EPCs (**Figures 1D, E**) no significant group*time existed (p>0.053), however the Cpep_und_ group had significantly lower group counts compared to both the non-diabetes controls and Cpep_high_ groups for these phenotypes (p<0.013).

Results were largely replicated when controlling for HbA1c, age or $\dot{V}O_{2peak}$ , with only the Cpep_high_ and control groups having significant increases from rest to post-exercise. When controlling for HbA1c, only HPCs lost the significant group*time interaction (p=0.072), despite group counts remaining significantly lower for Cpep_und_ compared to both Cpep_high_ and non-diabetes control groups. Controlling for $\dot{V}O_{2peak}$ did not alter the significant group*time interactions for any of the phenotypes. When controlling for age, EPCs also had a significant group*time interaction (p= 0.034).

When controlling for duration of diabetes between the high and undetectable residual beta-cell groups, group*time interactions remained for CXCR4^+^ HPCs (p=0.014), CXCR7^+^ HPCs (p=0.030) and CXCR7^+^ EPCs (p=0.007), but not for HPCs (p=0.060). Additionally, EPCs had a significant group*time interactions (p=0.020). Across all phenotypes, with duration of diabetes controlled at 19 years, Cpep_high_ had exercise-induced increases of 35-131%, whereas Cpep_und_ had no significant increase of between -1 to 9%.

## 4 Discussion

For the first time, we show that a high residual beta-cell function in type 1 diabetes is associated with intact exercise-induced increases in circulating HPC and EPC numbers, comparable to non-diabetes controls. However, in those with undetectable C-peptide, rapid mobilisation in response to exercise appears to be lost. This is despite similar HbA1c, age, and fitness between type 1 diabetes groups, all clinical characteristics that previously have been demonstrated to influence circulating counts (22, 26, 36). In combination with research demonstrating that post-islet transplantation patients have improved angiogenic function of EPCs compared to non-transplant controls (37), our data suggest that β-cell function in type 1 diabetes can influence angiogenic cells.

Acute exercise can mobilise EPCs into the circulation in healthy individuals, as well as improving the angiogenic function (38). Exercise-induced increases have been hypothesised as a surrogate marker for mobilisation in response to ischemic stressors. Specifically, reduced exercise-induced mobilisation of CD34^+^CXCR4^+^ HPCs is associated with increased CVD in patients with coronary artery disease (27). As the Cpep_und_ group had no significant exercise-induced increases, this may be a mechanism by which this group are at increased risk of future diabetes-related complications (3).

Despite HbA1c being similar between the patient groups at the time of testing, it is possible those with high residual beta-cell function have had reduced time spent in hyperglycaemia and improved glycemic variability (3, 10). Additionally, the lack of historic HbA1c determined over the duration of the diabetes is a limitation, as the high β-cell function group may have had reduced exposure to hyperglycaemia over time. Exposing angiogenic cells to hyperglycaemia *in vitro* results in increased apoptosis and a decrease in function, while improving HbA1c and glycaemic variability has been shown increased numbers of circulating cells (22). Hyperglycaemia also leads to increased vascular damage and disruption of bone marrow homeostasis, ultimately resulting in the exhaustion, depletion and inability to mobilise angiogenic cells from within the bone marrow (39).

It is possible that endogenous insulin and C-peptide secretion directly into circulation *via* the portal vein, may also offer some vasoprotection. *In vivo* studies demonstrate that insulin improves mobilisation and stimulates the angiogenic function of EPCs (40), while *in vitro studies* demonstrate C-peptide reduces hyperglycaemia-induced dysfunction in mature vascular endothelial cells (41) and stimulates ATP release from erythrocytes (42). Further investigation is needed to determine whether the small amounts of endogenous secretion from functioning beta-cells can be protective against diabetes complications.

Even when controlling for duration, circulating counts were higher and exercise-induced increases were only seen in those with high beta-cell function. However, it is important to recognise these results may still be influenced by accumulative years of exposure, with a longer duration of diabetes associated with reduced resting count (36) and our high C-peptide group having a significantly shorter duration. Despite this, some individuals within the high C-peptide group had type 1 diabetes for 20 years. As this was an exploratory study and as such was underpowered, this should be considered as a limitation. However, we hope this novel analysis will inform future studies exploring residual β-cell function and angiogenic cells, in particular focusing on the development of diabetes complications.

In summary, individuals with type 1 diabetes who have a high residual beta-cell function have an intact ability to increase circulating angiogenic cell numbers in response to exercise. It remains to be determined whether this is due to improved glycaemic control, clinical characteristics or the direct impact of endogenous insulin and C-peptide secretion upon the vasculature. Further investigation is needed to understand if these findings translate to reduced development of micro and macro-vascular complications.

## Data Availability Statement

The raw data supporting the conclusions of this article will be made available by the authors, without undue reservation.

## Ethics Statement

The studies involving human participants were reviewed and approved by HS HRA North East Tyne & Wear South Research Ethics (code:16/NE/0192) and Newcastle University Ethics Committees. The patients/participants provided their written informed consent to participate in this study.

## Author Contributions

GT recruited participants, designed study, researched data, wrote the manuscript. DW and MR designed study, researched data, wrote the manuscript. JAS recruited participants, designed study, provided clinical cover and reviewed/edited the manuscript. TM analysed samples and reviewed/edited the manuscript, AS processed data, reviewed/edited the manuscript. MC reviewed/edited the manuscript. KS and JHS contributed to data collection and reviewed/edited the manuscript. DW is the guarantor. All authors contributed to the article and approved the submitted version.

## Funding

This study was funded by the Diabetes Research and Wellness Foundation (SCA/OF/12/15) award to DW. Funding was also provided by philanthropic award to DW from the Francis James Bell Endowment Fund, Country Durham Community Foundation. The study funders was not involved in the design of the study; the collection, analysis, and interpretation of data; writing the report; and did not impose any restrictions regarding the publication of the report

## Conflict of Interest

The authors declare that the research was conducted in the absence of any commercial or financial relationships that could be construed as a potential conflict of interest.

## Publisher’s Note

All claims expressed in this article are solely those of the authors and do not necessarily represent those of their affiliated organizations, or those of the publisher, the editors and the reviewers. Any product that may be evaluated in this article, or claim that may be made by its manufacturer, is not guaranteed or endorsed by the publisher.
